# Prenatal Diagnosis and Obstetrical Challenges of a Massive Fetal Ascites Affecting One Fetus in a Dichorionic Diamniotic Twin Pregnancy: A Case Report

**DOI:** 10.7759/cureus.115939

**Published:** 2026-09-08

**Authors:** Aboubakr Benjilany, Saad Benali, Mly Mehdi Elhassani, Jaouad Kouach

**Affiliations:** 1 Department of Obstetrics and Gynecology, Military Hospital of Instruction Mohammed V, Rabat, MAR

**Keywords:** dichorionic diamniotic twin pregnancy, fetal ascites, fetal hydrocele, fetal weight estimation, obstetric management, prenatal diagnosis

## Abstract

Fetal ascites is a rare prenatal condition; it may result from a wide range of underlying etiologies, including structural anomalies, genetic disorders, congenital infections, and metabolic diseases. Despite considerable advances in prenatal investigations, etiology remains unidentified in some cases. Massive fetal ascites may also complicate obstetric management by distorting fetal biometric measurements and influencing delivery planning.

We report the case of a 34-year-old woman with a spontaneously conceived dichorionic diamniotic twin pregnancy. At 24 weeks of gestation, massive fetal ascites associated with bilateral hydrocele was diagnosed in a male twin. Maternal infectious investigations were negative except for low-titer Epstein-Barr virus (EBV) IgG positivity. The patient presented with threatened preterm labor at 30 weeks of gestation. Ultrasound estimated the affected fetus to weigh approximately 3.3 kg because of marked abdominal distension secondary to ascites, whereas the actual birth weight was 2.2 kg. The infant died approximately one hour after birth, while the co-twin survived following admission to the neonatal intensive care unit.

This case illustrates the diagnostic challenges posed by massive fetal ascites, even after comprehensive prenatal evaluation. It also highlights its obstetrical implications, particularly the impact on fetal weight estimation and perinatal management in twin pregnancies.

## Introduction

Fetal ascites is a rare prenatal circumstance, defined by abnormal accumulation of free fluid within the fetal peritoneal cavity. It may occur as an isolated ultrasound finding or as a manifestation of fetal hydrops [1]. Etiologies are diverse and include congenital infections, structural gastrointestinal or genitourinary anomalies, chromosomal abnormalities, metabolic disorders, and fetal anemia [2]. Despite considerable advances in prenatal imaging investigations, the underlying cause remains undetermined in a substantial proportion of cases [3].

The prognosis of fetal ascites depends largely on underlying etiology, associated anomalies, and gestational age at diagnosis [3]. In addition, massive fetal ascites may complicate prenatal assessment by distorting fetal biometric measurements and influencing obstetric management.

The occurrence of massive fetal ascites, affecting only one fetus in a dichorionic diamniotic twin pregnancy, is an unusual clinical presentation. We report a case of massive fetal ascites, associated with bilateral hydrocele in one twin, for which no prenatal etiology could be identified. The case was characterized by marked overestimation of fetal weight on ultrasound and particularly challenging perinatal management. It highlights diagnostic challenges of massive fetal ascites and its obstetric implications in the unique setting of a dichorionic diamniotic twin pregnancy.

## Case presentation

A 34-year-old gravida 2, para 1 woman, with no relevant medical, surgical, or family history, presented with a spontaneously conceived, dichorionic diamniotic twin pregnancy. The previous pregnancy had resulted in an uncomplicated term vaginal delivery of a healthy male infant. Her blood group was O Rh-positive.

Prenatal care consisted of only two antenatal visits and two obstetric ultrasound examinations. Dichorionic diamniotic twin pregnancy was confirmed at 12 weeks and 4 days of gestation.

At 24 weeks of gestation, an ultrasound examination revealed intra-abdominal fetal ascites associated with a bilateral hydrocele in the male twin (Twin 1). No additional abnormalities were documented in the available ultrasound report. The female co-twin (Twin 2) showed no reported abnormalities.

Maternal investigations performed to determine etiology included serological testing for syphilis, toxoplasmosis, rubella, hepatitis viruses (B and C), cytomegalovirus, parvovirus B19, human immunodeficiency virus (HIV), and herpes simplex virus. All results were negative. The only abnormal finding was low-titer Epstein-Barr virus (EBV) IgG positivity. Repeat serological testing was scheduled three weeks later to clarify its significance but could not be performed because of preterm delivery. Consequently, a possible role of EBV could not be confirmed. Neither amniocentesis nor non-invasive prenatal testing was proposed during the pregnancy.

At 27 weeks of gestation, the patient was admitted with generalized pruritus, associated with elevated liver enzyme levels. A diagnosis of intrahepatic cholestasis of pregnancy was established, and appropriate symptomatic treatment was initiated.

At 30 weeks of gestation, she was admitted for preterm labor, without premature rupture of membranes. On examination, she was afebrile and hemodynamically stable. Cervical assessment revealed 80% effacement with approximately 2 cm dilation, and Twin 1 was in breech presentation.

Laboratory investigations, including a complete blood count, C-reactive protein, urine culture, and liver function tests, showed a normal blood count, negative inflammatory markers, sterile urine culture, and persistently elevated but stable liver enzyme levels.

Ultrasound demonstrated progression of the ascites in Twin 1, resulting in marked abdominal distension, with persistent bilateral hydrocele. The estimated fetal weight was approximately 3300 g. Umbilical artery and middle cerebral artery Doppler findings were within normal limits in both fetuses. Both fetal bladders were visualized and appeared normal, and amniotic fluid volumes were normal for both twins. No ultrasound or Doppler findings suggestive of twin-to-twin transfusion syndrome were identified. Twin 2 was also in breech presentation, with an estimated fetal weight of approximately 1490 g (appropriate growth for gestational age), a normal-appearing placenta, and normal amniotic fluid volume. A dedicated fetal echocardiographic examination could not be performed because of the urgent clinical context and subsequent preterm delivery; however, no structural cardiac abnormality had been identified on the second-trimester ultrasound examination.

Despite tocolytic therapy and corticosteroids for fetal lung maturation, cervical dilation progressed to 5 cm. An emergency cesarean section was therefore performed because of progressive preterm labor in the setting of a dichorionic diamniotic twin pregnancy, breech presentation of Twin 1, and fetal abdominal distension secondary to massive ascites.

Twin 1, a male infant, was delivered by breech extraction. The Apgar score was 3 at 1 minute. Clinical examination revealed marked abdominal distension due to massive ascites, associated with bilateral hydrocele, without any obvious external congenital malformations. His birth weight was 2150 g. Despite prolonged resuscitation, the newborn died approximately one hour after birth (Figure 1).

**Figure 1:**
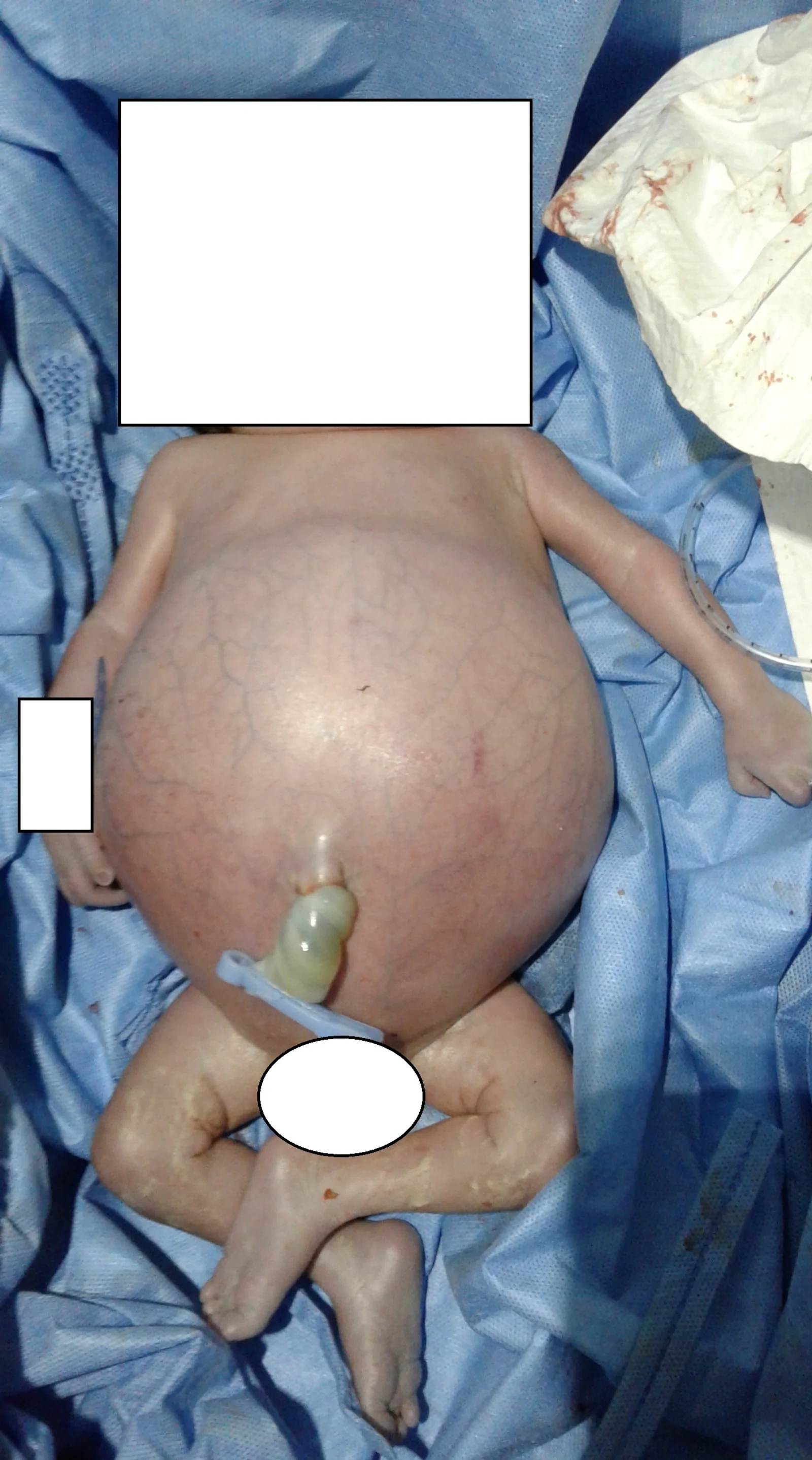
Newborn Twin 1 (male) presenting with massive ascites.

Twin 2, a female infant, was also delivered in breech presentation. Her birth weight was 1400 g, and Apgar scores were 7 at 1 minute and 8-9 at 5 minutes. She developed respiratory distress related to prematurity and was admitted to the neonatal intensive care unit.

During cesarean delivery, the dichorionic diamniotic nature of the twin pregnancy was confirmed intraoperatively. Placenta macroscopic examination revealed a grape-like appearance. Samples were submitted for histopathological, bacteriological, and virological analyses; however, the final results were unavailable at the time this manuscript was prepared.

## Discussion

Isolated ascites is diagnosed by the presence of fluid around the liver, spleen, intestine, extrahepatic portion of the umbilical vein, falciform ligament, or greater omentum. When fetal ascites is first identified on ultrasound, a repeat examination one week later is recommended to assess whether hydrops fetalis has developed. If the fluid collection remains confined to the abdomen, the probability of progression to hydrops is considerably lower [1].

Despite advances in prenatal investigations, determining the etiology of fetal ascites remains complex in a significant proportion of cases, even with a rigorous and systematic diagnostic approach [2,3]. Our case illustrates several challenges, including the occurrence of massive isolated fetal ascites in a single fetus of a dichorionic diamniotic twin pregnancy, its obstetric implications, and the persistence of etiological uncertainty despite a thorough prenatal workup.

Isolated fetal ascites is more commonly associated with an underlying intra-abdominal abnormality than with a systemic disorder. Obstructive uropathy represents the principal cause, whereas gastrointestinal pathologies account for approximately 20% of cases [2-4]. Among these, meconium peritonitis, usually resulting from intestinal obstruction, is the most frequently reported etiology [5]. Other reported etiologies include congenital infections, chromosomal abnormalities, fetal anemias, metabolic disorders, and lymphatic abnormalities [1,6].

Evaluation of fetal ascites should begin with an exhaustive ultrasound examination to identify or exclude associated fetal anomalies. Maternal serological testing for infections, including hepatitis viruses, rubella, syphilis, toxoplasmosis, parvovirus, cytomegalovirus, varicella, and herpes simplex virus, should also be performed [1,7]. In addition, the Rhesus factor and maternal antibody titers should be determined. Fetal karyotyping by amniocentesis should also be considered, given that chromosomal abnormalities have been reported in up to 15% of cases [7,8]. In addition to cytogenetic testing, investigations for possible fetal infection, including TORCH serology and pathogen-specific IgM/IgG antibodies, as well as prenatal screening for inherited metabolic disorders, may provide valuable information [1,7]. Similar investigations can also be performed using fetal blood samples. Fetal paracentesis may be considered to assess the presence of meconium, measure protein and lymphocyte levels, and perform TORCH testing and fetal karyotyping [1].

In our case, fetal ascites affected one fetus, while the co-twin was morphologically normal. The ascites was associated with bilateral hydrocele, without any other morphological abnormalities. Although maternal serology revealed weak positivity for anti-EBV IgG antibodies, this result should be interpreted with caution, as anti-EBV IgG antibodies reflect past rather than recent infection [9]. Furthermore, the absence of repeated maternal serology, placental histopathological findings, and neonatal diagnostic examinations prevented the establishment of a causal link between possible EBV infection and fetal ascites [9]. The discrepancy between estimated fetal weight by ultrasound and actual birth weight highlights the limitations of conventional fetal weight estimation in major fetal abdominal pathology [2,3].

Thoracic compression, caused by severe intra-abdominal ascites before 24 weeks of gestation, may result in pulmonary hypoplasia; however, the benefit of fetal paracentesis in this setting remains unclear [1,10]. Fetal echocardiography should also be performed to exclude structural cardiac abnormalities or fetal arrhythmias [1]. In a series of 12 cases of isolated fetal ascites reported by El Bishry, 10 pregnancies resulted in viable outcomes. Among these, two infants were diagnosed postnatally with gastrointestinal abnormalities that required surgical treatment: one had ileal atresia, and the other had bowel atresia associated with a large pseudocyst [7]. Two fetal losses occurred in cases diagnosed before 20 weeks of gestation, one of which was associated with fatal laryngeal atresia [7]. Similarly, Favre et al. reported favorable outcomes among cases of isolated idiopathic fetal ascites, in which no underlying cause was identified during the perinatal period [3].

Parents should be informed of the etiologies and differential diagnoses' wide range, the prognosis uncertainty, and the limitations of prenatal testing [7,11,12]. Ultimately, management should consider gestational age, etiology, course of fetal ascites, associated fetal anomalies, and the overall obstetric context [12].

## Conclusions

Isolated fetal ascites is a rare prenatal circumstance that should be distinguished from hydrops fetalis. It encompasses a wide range of possible etiologies and differential diagnoses. A comprehensive prenatal evaluation is essential to identify the underlying cause, as most cases are associated with additional fetal abnormalities. Nevertheless, the etiology may remain unidentified, despite a thorough diagnostic workup. Overall, isolated fetal ascites is associated with a more favorable perinatal outcome than hydrops fetalis.
